# Abundance, Distribution and Population Trends of Waterbirds in the Usangu Wetland: A Biodiversity Hotspot Threatened by Human Activities

**DOI:** 10.1002/ece3.73379

**Published:** 2026-04-08

**Authors:** Amani Chaula, Safari Ignas, Anibariki Ngonyoka

**Affiliations:** ^1^ Department of Biology, College of Natural and Mathematical Sciences University of Dodoma Dodoma Tanzania

**Keywords:** abundance, and Usangu wetland, distribution, population trend, water birds

## Abstract

Waterbirds play essential ecological function contributing to maintenance of species diversity, pest control, seed dispersal, and vectors for invertebrates. Approximately one‐third of the waterbird assemblage inhabits wetland environments. However, waterbirds and their habitats are threatened by human activities capable of disrupting ecosystem. This study was conducted in Usangu Wetland Important Bird Areas to investigate the abundance, distribution, and population trends of wild avian species. Bird counting survey and questionnaire were conducted from to elicit information about abundance and distribution, and bird population trend respectively between March and May, 2022. Bird counts were conducted using 2.5 km line transects at three sites. Each site had six transect replicates to estimate relative abundance and distribution. Structured and key informant interviews were used to elicit trends of bird populations over the past 20 years and their influencing factors. Data were analyzed using R‐studio version 4.0.3. Furthermore, content analysis was employed to scrutinize perceived population trends. The study cataloged a total of 34 distinct species of waterbirds. The most prevalent species included lesser moorhen ( *Gallinula angulata* ) 47.56%, Fulvous Whistling Duck ( *Dendrocygna bicolor* ) 12.01%, and Glossy ibis ( *Plegadis falcinellus* ) 10.15%. The distribution patterns of waterbirds were determined by habitat type, geographical location, farm characteristics, and the specific rice scheme type, with a greater concentration in rice plantations and outgrowers rice schemes. Local accounts underscored a declining trend in waterbird populations over past 20 years due to illegal hunting and habitat conversion. Usangu wetland has a higher abundance of water birds with disproportionate distribution bias in rice farms. In spite of rice farms suitably holding a high assemblage of water birds, its sustainability is subject to owners' decision, vegetation cover changes after rice harvest characterized by illegal hunting.

## Graphical abstract

Waterbirds play vital ecological roles in wetland ecosystems. This study reports variations for 34 species of waterbirds across study sites of Usangu Wetland abundance with distribution bias in rice farms. The waterbird population trend is declining because of habitat alteration and hunting hence conservation measures should be taken to enhance biodiversity integrity.

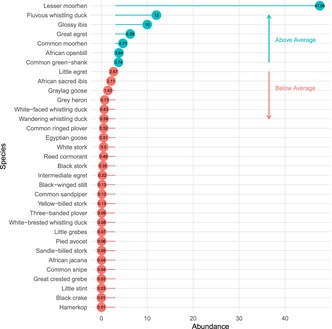

## 1 Background Information

Waterfowl species occupy indispensable ecological niches in aquatic ecosystems, contributing significantly to the maintenance of other species, regulation of pest populations, dispersion of seeds, predation, nutrient cycling, and serving as early indicators of potential disease outbreaks (Ref). Although one‐third of waterbirds water birds the wetlands apart from rivers, lakes, ponds and coastal areas, currently the habitats and waterbirds are threatened by human activities potentially affecting ecosystem integrity (Amano et al. 2020). In this research study, waterbirds are defined as birds belonging to various avian taxonomic groups with adaptive features to live in aquatic environments, which include webbed feet, waterproof feathers, and long bills for foraging.

Wetlands are not only important for waterbirds but also for up to one‐fifth of the world's bird species directly or indirectly depend seasonally or permanently on wetlands for feeding, breeding, resting, and overwintering (Dudgeon et al. 2006). In addition, wetlands provide staging grounds for migratory species of birds. Examples of water birds include wildfowl, coots, waders, herons, cormorants, flamingos, and grebes (Rannestad et al. 2015). However, wetlands exhibit disproportionate habitat alteration in the world than other aquatic habitats, hence negatively affecting the abundance and distribution of water birds (Maneas et al. 2020). About half of water bird species are declining, 4% have gone extinct, about 17% are increasing, and only one‐third are stable (Amira et al. 2018). Furthermore, there is a concerning projection that 12% of bird species will become extinct in the next 10 to 100 years Consulting (2017).

Composition of bird communities within wetland environments is subject to the influence of a multitude of environmental determinants; such as water depth, physical and chemical properties of water, habitat availability, degree of isolation, intricate species interactions, and population dynamics (Haig et al. 2019). Abundance of species in a location is also determined by local and regional processes (Haig et al. 2019). Regional processes include long‐distance dispersal, speciation, or local extinction; local processes include predation, parasitism, competition, and disturbances (Cintra 2012; Bennett et al. 2006). Consequently, locally abundant species tend to have larger distributions and the replacement of individuals may be the result of immigration from the regional species pool in addition to species birth rate (Haig et al. 2019). In island biogeography, the abundance of water birds in patchy habitats of the wetlands may correlate positively with area size and decrease with distance from the source (Dm 2005).

About 10% of Tanzania's land surface area is covered by various wetlands (Dudgeon et al. 2006). Tanzania ratified the Ramsar Convention in 2000 and at present has four Ramsar sites: Rufiji‐Kilwa Marine Ramsar Site, Kilombero Valley Flood Plains, Lake Natron Basin, and Malagarasi‐Moyovozi wetlands, while other wetlands fall within the jurisdiction of other protected areas such as national parks and game reserves. Still, up to 17,133.3 Km^2^ of wetland Important Bird Area (IBA) in Tanzania contains the lowest category of protection where over one‐third of the area is comprised of wetlands (Cintra 2012). Wetlands act as carbon sinks and local foci for biodiversity (Bennett et al. 2006). However, apart from ecological benefits, wetlands are important sources of clean water and firewood for the communities. They have immense potential for agricultural output (Hagen et al. 2012) and are key avenues for a wide range of socio‐cultural activities (Sritharan and Burgess 2012).

Usangu Wetland harbors diverse resident birds including the endemic cuckoo namely: *Centropus cpreicaudas*, *C. superciliosus* , *C. senegalensis*, and *C. grillii*, different species of ducks, storks, moorhens, egrets and seasonal migratory birds such as white stork. The wetland is endowed with its alluvial soil, water availability, abundant grazing opportunities, forested areas, and wild animals. However, recent research indicates a profound transformation of the wetland's landscape, characterized by the conversion of grasslands and woodlands into cropland and pasture (Franks et al. 2004). This land‐use change has led to the reduction of wetland areas over the past 30 years, hence an increase in bare land (Kingsford et al. 2016; Ma et al. 2009). Despite these ongoing transformations that have altered the structural dynamics of the wetland, there remains a notable absence of comprehensive scientific literature detailing the repercussions of anthropogenic pressures on water bird populations. Therefore, the primary objective of this paper is to address this critical research gap by conducting an ecological study focused on answering three questions: (1) What is the relative abundance of waterbirds in Usangu Wetland? (2) What are the factors affecting the distribution of waterbirds in Usangu Wetland? and (3) What are the perceived population trends of waterbirds over 20 years ago in the Usangu Wetland? This research endeavors to contribute valuable insights into the impact of human activities on the avian populations inhabiting this vital ecosystem.

## 2 Methodology

### 2.1 Study Area

**FIGURE 1:**
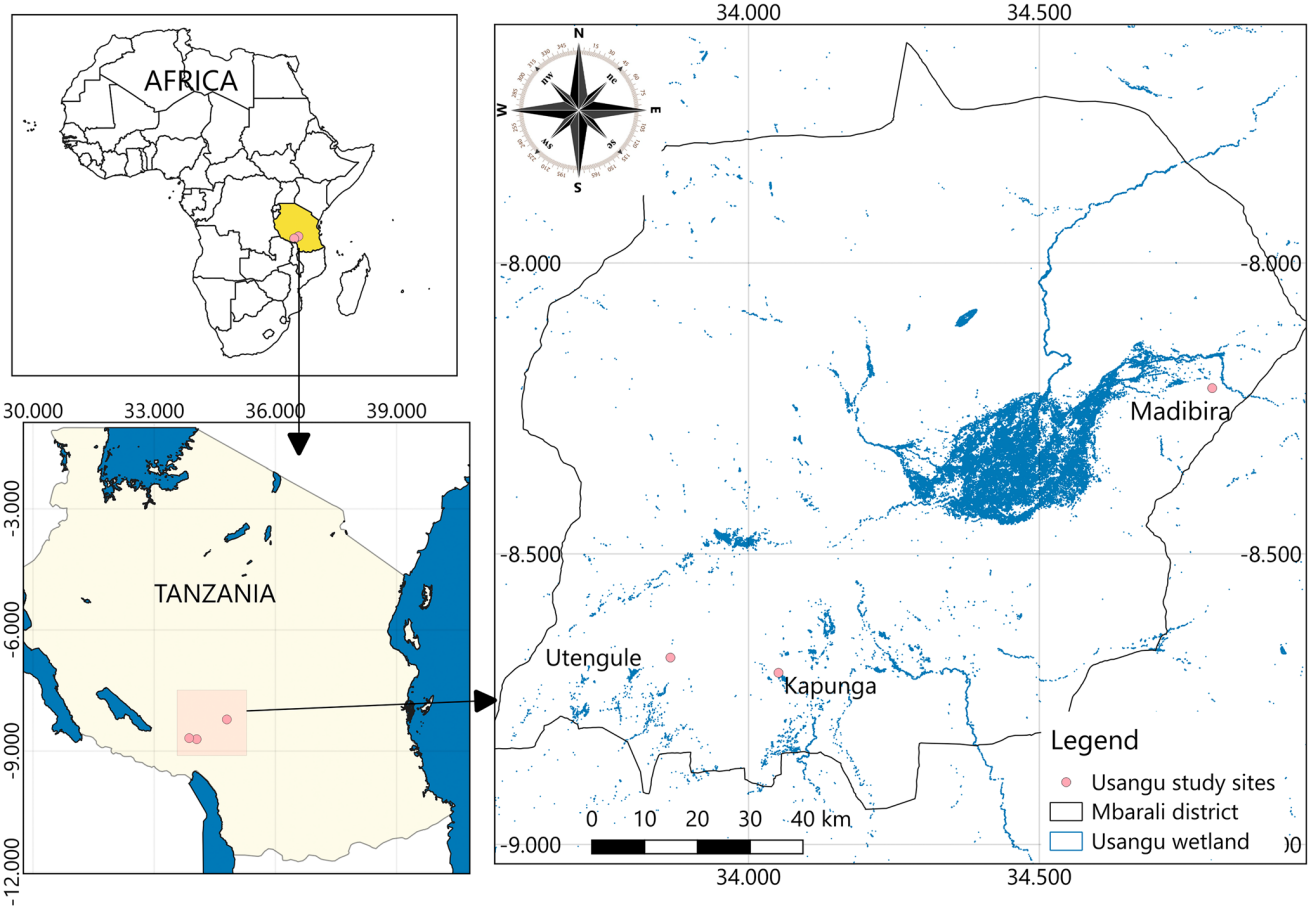
Map of Usangu wetland showing the location study sites.

Usangu Wetland is situated within the larger Usangu basin, positioned between latitude 7°41′ and 9°25′ south and longitude 33°40′ and 35°40′ East in the southwestern region of Tanzania (Figure 1). Covering an area of approximately 20,800 km^2^, the Usangu basin encompasses the Usangu upper catchment, alluvial fans, the wetland itself, the riparian stretch within Ruaha National Park, and the Mtera/Kidatu hydroelectric system (Franks et al. 2004). The Usangu wetland, located at an altitude of 1100 m above sea level, constitutes roughly one‐tenth of the entire basin. It receives an average annual rainfall of 700 mm, predominantly falling from December to March. Additionally, the wetland benefits from water drainage originating in a mountainous catchment situated to the south, characterized by altitudes reaching 3000 m and an annual rainfall of 1000 to 1500 mm. This abundant water supply sustains irrigation schemes across the plains, including the wetlands in the northern region (O'Connell 2000; Mwakalila 2011). The livelihood of the people depends on livestock keeping and agriculture; hence mostly settled around alluvial plains. The alluvial fans, in turn, give way to extensive wetland comprising seasonally flooded grassland and a small area of permanent swamp. The ecology of Usangu is recognized as an important bird and biodiversity area (IBA), which plays a key role in supporting the abundance of diverse birds and, much more importantly, water birds. In recent years, the wetlands have experienced a rapid conversion of wetlands to rice irrigated schemes and outgrower's rice farming, compromising the habitats of the waterbirds.

### 2.2 Study Design

This research employed a cross‐section study design where the data on bird count survey and the questionnaire survey on waterbirds trends in the study area were collected once at a time. Estimates of relative abundance of waterbirds used line transects length of 2.5 km and 500 m width. The transects traversed various habitat characteristics, farm characteristics, scheme types, and distances from various land uses. The description for habitat characteristics includes rice farms, composed of paddy at different growth stages; maize farms, composed of grown maize at different stages of growth; grassland, composed of grassy vegetation in the uncultivated area; and water bodies, covered by water throughout the study period. Rice farm characteristics consist of rice reproduction phase, vegetation growth, and preparation stage. In addition, distances from waterbirds sightings to water bodies, distance from farmland, settlement, grazing land, and ponds were recorded for further analysis. While rice schemes were categorized into plantation schemes and out‐grower schemes. The interview method was used to elicit information on trends of waterbird population from randomly selected 343 households and 16 key informant interviews.

### 2.3 Data Collection

Ecological survey on relative abundance of water bird was carried out in three selected sites of Usangu wetland namely: Kapunga, Madibira and Utengule of the flooded plain during the wet season in March and May of 2022. 6 transects each with 2.5 km length and inter‐transect distance of 1 km were established for waterbirds counting in each site. Birds were counted within an eye visible range of 100 m width during 9:00–11:00 and 15:00–16:00. During counts, each sighting of water birds, habitat features, and habitat characteristics were geo‐referenced and recorded. Binoculars with power and field of view 7 × 35 were used to observe morphometric features of birds for proper identification birds of East Africa field guide. In addition, distance of bird sightings from various land uses were measured using Google Earth. Data on the status and historical population trend of water birds were elicited through structured questionnaire from 343 respondents with informed consent. Respondents sampled from three study sites Kapunga, Madibira and Utengule were 112, 130 and 101 respectively. The respondents were randomly selected from the list provided by local government officials to participate in structured interview survey between March to May 2022. The sample size for this study was determined based on the methodological prescription of Taherdoost (Kitalika and Mlengule 2021). The formula used to calculate the number of respondents: $n=\frac{Zc^{2}P\left(1-P\right)}{E^{2}}$, where *Z*_*c*_‐the value corresponding to level of confidence required set at 95% confidence interval (1.96), P‐Percentage of people with knowledge of waterbirds ecology, E‐Percentage to commit maximum error and *n* = sample size. Prior to data collection, the structured questionnaire was pre‐coded, with response options assigned numerical values to facilitate quantitative analysis of household perceptions regarding waterbird population decreasing trends over the past 20 years. In order to triangulate information gathered from household questionnaire survey 16 key informants who were selected based on their job position in the villages or district. The list included 3 village chairmen, 1 district game officer, 3 agricultural officers and 9 community leaders lived in the area for long time. The key informants provided detailed insights on abundance of water birds, timing of migratory water birds in the area, habitat change over time in the study area.

### 2.4 Ethical Consideration

Ethical approval for this research was granted by the Institutional Research Review Committee at the University of Dodoma (Reference: MA.84/261/02/‘A’). Prior to enrollment, all participants were thoroughly briefed on the study and gave their voluntary consent. Confidentiality was maintained throughout, participants could exit the study at any time without penalty, and all data were anonymized and stored securely according to institutional guidelines.

### 2.5 Data Analysis

The quantitative data on relative abundance, distribution and habitat characteristics of water birds species were cleaned and displayed in tables and charts using ggplot 2 in R‐studio software version 4.4.0. Regression analysis was used to analyze the abundance of waterbirds on habitat characteristics and distribution. Mean comparison of the abundance of birds among sites and regression coefficients were considered significant when *p* < 0.05. Data on socio‐demographic characteristics and household perception of water bird population trend were cleaned, coded, and entered into Statistical Package for Social Sciences (SPSS) version 26 (Comber et al. 2021). Frequencies and percentage composition of the demographic characteristics and population trend were presented in tables and analyzed using the Chi‐square test. The qualitative data obtained through Key informant interview (KI) were analyzed using Content Analysis (CA), which assisted in categorizing the data collected into several subjects connected to the study topic and presenting them in the form of quotations as conclusions or information to encourage discussion.

## 3 Results

### 3.1 Relative Abundance of Water Birds in Usangu Wetland

**FIGURE 2:**
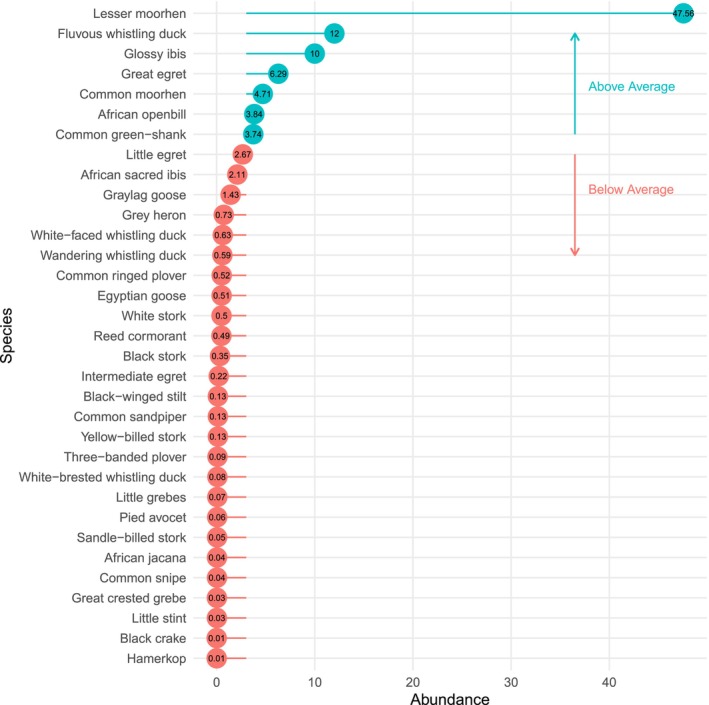
The relative abundance of waterbird species in relation to the average in Usangu wetland surveyed in March–May 2022.

The study documented 34 species of waterbirds. The most abundant species were *Gallinula angulata* (47.56%), *Dendrocygna bicolor* (12.01%), and *Plegadis falcinellus* (10.15%). The remaining 31 water bird species had low abundance levels, which cumulatively contributed 30.28% (Figure 2).

### 3.2 Distribution of Water Birds

Distribution of water birds across Madibira, Kapunga, and Utengule three study sites. 16 out of 34 waterbird species were confirmed to occupy all the three study sites in Usangu wetland. The black crake ( *Amaurornis flavirostra* ) was only found in Madibira. Likewise, white‐faced whistling ducks ( *Dendrocygna viduata* ), graylag goose ( *Anser anser* ) and hamerkop ( *Scopus umbretta* ) were found in Kapunga. and little grebes ( *Tachybaptus ruficollis* ) was only found in Utengule, and 14 species were recorded in two sites either of the three sites. However, the majority of waterbird species were locally documented at Kapunga, compared to Utengule and Madibira (Table 1).

**TABLE 1:** Distribution of water bird by species across the three study sites.

S/N	Water bird species	Scientific name	Madibira	Utengule	Kapunga
1	African jacana	*Actophilornis africanus*	+		+
2	African openbill	*Anostromus lamelligerus*	+	+	+
3	African sacred ibis	*Threskiornis aethiopicus*		+	+
4	Black crake	*Amaurornis flavirostra*	+		
5	Black stork	*Ciconia nigra*	+	+	+
6	Black winged stilt	*Himantopus himantopus*	+	+	+
7	Common greenshank	*Tringa nebularia*	+	+	+
8	Common moorhen	*Gallinula chloropus*	+		+
9	Common ringed plover	*Charadrius hiaticula*	+		+
10	Common sandpiper	*Actitis hypoleucos*	+	+	+
11	Common snipe	*Gallinago gallinago*		+	+
12	Egyptian goose	*Alopochen aegyptiaca*	+	+	+
13	Fulvous whistling ducks	*Dendrocygna bicolor*	+	+	+
14	Glossy ibis	*Plegadis falcinellus*	+	+	+
15	Great crested grebe	*Podiceps cristatus*			+
16	Great egrets	*Ardea alba*	+	+	+
17	Gray heron	*Ardea cinerea*	+	+	+
18	Graylag goose	*Anser anser*			+
19	Hamerkop	*Scopus umbretta*			+
20	Intermediate egrets	*Ardea intermedia*		+	+
21	Little egrets	*Egretta garzetta*	+	+	+
22	Little grebes	*Tachybaptus ruficollis*		+	
23	Little stint	*Calidris minuta*	+	+	
24	Marabou stork	*Leptoptilos crumenifer*	+	+	+
25	Pied Avocet	*Recurvirostra avosetta*		+	+
26	Reed cormorant	*Microcarbo africanus*		+	+
27	Saddle billed storks	*Ephippiorhynchus senegalensis*		+	+
28	Three banded plovers	*Charadrius tricollaris*	+		+
29	White‐breasted whistling ducks	*Dendrocygna autumnalis*	+	+	
30	White‐faced whistling ducks	*Dendrocygna viduata*			+
31	White stork	*Ciconia ciconia*	+	+	+
32	Wondering whistling ducks	*Dendrocygna arcuata*	+	+	+
33	Yellow billed storks	*Mycteria ibis*	+	+	+
34	Lesser moorhen	*Gallinula angulata*	+	+	+

There was uneven distribution where most species were highly aggregated in Kapunga accounting for up to 83% of the total water birds recorded (*χ* ^2^ = 31,483, d.f = 2, *p* < 0.0001).

Data on birds' distribution was also reported during KII as one of the informants said that:

There is non‐uniform assemblage of water birds in the sites of Usangu wetland because the size and number of natural water and man‐made water infrastructure that provide suitable habitats is different across the wetland. (KI, male 38, community leader)

In addition, there was a marginal decline in the number of bird sightings along the distance gradient from water bodies, which shows suitability of water ponds as habitat for the waterbirds. Conversely, distance from settlements, grazing land and farming lands were not statistically significant (Table 2). The vegetative phase of rice growth contained fewer water birds than the reproductive phase (Table 2). In addition, small waterbirds such as pied avocets, common ringed plovers, three banded plovers, little stints, and egrets were observed in the sites during the vegetative phase, whereas, lesser moorhens, colonies of fulvous whistling ducks, and common moorhens were extremely abundant during the reproductive phase (grown rice). Other bird species, such as gloss ibis, African open‐billed storks, gray herons, and marabou storks, were primarily observed and recorded in the vegetative phase boundaries between rice paddies. However, the abundance of water birds differed considerably among habitat types compared to the rice habitat (Table 2).

**TABLE 2:** Multiple linear regression of water bird distribution along distance gradient, rice growth stage and habitat type.

Number of birds observed	Coefficients	Standard error	*t*‐value	*p*	Lower 95% CI	Higher 95% CI
Distance from land cover						
Distance from water bodies	−0.037	0.019	−1.95	0.052	−0.075	0.0003
Distance from settlements	0.005	0.010	−0.47	0.642	−0.0245	0.0151
Distance from grazing land	0.010	0.005	1.84	0.066	−0.001	0.0205
Distance from farm	0.0448	0.035	1.28	0.201	−0.024	0.1136
Rice growth stage	—	—	—	—	—	—
Reproductive phase	Ref	—	—	—	—	—
Vegetative growth	−154.760	30.9809	−5.25	0.000	−223.636	−101.8830
No field paddy	−164.648	25.386	−4.16	0.000	−155.533	−55.7634
Habitat type						
Rice farm	Ref	Ref	—	—	—	—
Grassland	−71.316	26.275	−2.52	0.012	−126.878	−15.7533
Maize farm	−61.905	29.142	−2.12	0.034	−119.170	−4.6397
Water pond	−70.595	27.810	−2.54	0.011	−125.243	−15.9473
Constant	179.478	31.642	5.67	0.000	117.300	241.6560

*Note:* Ref denoted reference variable utilized for comparative analysis with other variables belonging to the same category.

In addition, when comparing the average difference of water birds in cultivated lands differ significantly from uncultivated lands across study sites (Table 3). One of the key informants reported a more or less similar finding that:

Normally water birds prefer cultivated areas are compared to uncultivated ones. The reason for such inclination is because rice cultivated farms resembles with natural sites with the added benefit of irrigated areas maintaining wet conditions even during dry seasons. (KI, male 45, village leader)

**TABLE 3:** Linear regression of water bird distribution in relation to cultivation status, location and scheme type.

Independent variables	Coefficients	Standard error	*t*‐value	*p*	Lower 95% CI	Higher 95% CI
Cultivation status						
Cultivated	Ref	—	—	—	—	—
Uncultivated	−42.102	10.086	−4.17	0.000	−61.923	−22.282
Location						
Kapunga	Ref	—	—	—	—	—
Madibira	−105.947	18.407	−5.76	0.000	−142.116	−69.777
Utengule	−96.611	17.857	−5.41	0.000	131.699	−61.523
Constant	121.575	18.061	6.73	0.000	86.085	157.067
Scheme type						
Outgrowers	Ref	—	—	—	—	—
Irrigation	176.724	27.501	6.43	0.000	122.482	230.967
Constant	11.653	1.485	7.84	0.000	8.723	14.583

*Note:* Ref denoted reference variable utilized for comparative analysis with other variables belonging to the same category.

Furthermore, the results show abundance of water birds in outgrower's rice farms is significantly lower than rice‐irrigated scheme (Table 3).

### 3.3 Perceptions of Local Communities on Water Birds' Population Trend

349 local community members comprised of 64.4% males and 35.6% females were recruited to participate in the study (Table 4). The age of the participants ranged from 18 to 70 years with a mean of 33.54 and a standard deviation of 13.02 years. 72% (*n* = 247) were from 18 to 40 years. The majority of respondents constituted 77.84% (*n* = 267) and reported having received primary and secondary education. The study shows local communities are engaged in hunting of wild animals including birds 36.44% (*n* = 125), farming 30.32%, entrepreneurship 18.1%, and hunting wild birds 18.8% (Table 4).

**TABLE 4:** Respondents' socio‐demographic profile across the three study sites.

Variable	Categories	Sites			
		Kapunga no. (*n* %)	Madibira no. (*n* %)	Utengule no. (*n* %)	Total no. (*n* %)
Sex	Female	35 (31.25)	62 (47.69)	25 (24.75)	122 (35.6)
	Male	77 (68.75)	68 (52.31)	76 (75.25)	221 (64.4)
Age	11–20	61 (54.46)	8 (6.15)	2 (1.98)	71 (20.7)
	21–30	31 (27.68)	42 (32.31)	33 (32.67)	106 (30.9)
	31–40	6 (5.36)	36 (27.69)	27 (26.73)	69 (20.12)
	41–50	10 (8.93)	17 (13.08)	30 (29.70)	57 (16.62)
	51–60	3 (2.68)	23 (17.69)	9 (8.91)	35 (10.20)
	61–70	1 (0.89)	4 (3.08)	0 (0.0)	5 (1.46)
Education level	Informal	7 (6.25)	23 (17.69)	6 (5.94)	36 (10.5)
	Primary	48 (42.86)	38 (29.23)	46 (45.54)	132 (38.48)
	Secondary	52 (46.43)	43 (33.08)	40 (39.60)	135 (39.36)
	Tertiary	5 (4.46)	26 (20.0)	9 (8.91)	40 (11.66)
Occupation	Hunters				
	Entrepreneurs	76 (67.86)	39 (30.0)	10 (9.90)	125 (36.44)
	Farmers	6 (5.36)	18 (13.85)	35 (34.65)	59 (17.20)
	Civil servant	17 (15.18)	45 (34.62)	42 (41.58)	104 (30.32)
	Livestock	4 (3.57)	9 (6.92)	5 (4.95)	18 (5.25)
	Keepers	0 (0.00)	1 (0.77)	3 (2.97)	7 (1.17)
	Unemployed	9 (8.04)	18 (13.85)	6 (5.94)	33 (9.62)

Furthermore, the study reveals that sex, age, site, marital status, and occupation had a significant association among the factors affecting respondents' perception of population trends of water birds (Table 5). Males perceived a higher decline in waterbird population trends than females, whereas respondents aged 21–30 and 31–40 perceived a higher decline in waterbirds compared to other age categories (Table 5). Hunters and farmers perceived a higher decline in waterbird population trends compared to other occupations. Participants with either primary or secondary education perceived that the decline of waterbirds was higher compared to informal and college education (Table 5). Respondents from Kapunga Madibira perceived a higher decline in waterbird population trends compared to Utengule (Table 5). Similarly, key informant interviews had similar views on the decreased population trend of water birds, as one of the participants said that:

Over the years, the number of birds over time has been decreasing where in the past we used to see birds covering the wetland like clouds. However, it is seldom to find such kinds of flock covering the sky. (KI, male 50, rice farmer)

**TABLE 5:** Factors influencing respondent's perception on the trends of waterbird abundance in Usangu Wetlands over the past 20 years.

Variable	Categories	Is the trend of waterbird populations in your area decreasing over the past 20 years?			df	Chi‐square *p*
		Yes no. (*n*%)	No no. (*n*%)	Total		
Sex	Female	85 (40.48)	37 (27.82)	122 (35.57)	1	5.6918 (*p* = 0.017)
	Male	125 (59.52)	96 (72.18)	221 (64.43)		
Marital status	Married	78 (37.14)	97 (72.93)	175 (51.02)	1	41.7377 (*p* < 0.0001)
	Unmarried	132 (62.86)	36 (27.07)	168 (48.98)		
Age	11–20	67 (31.90)	4 (3.02)	71 (20.70)	5	59.3621 (*p* < 0.0001)
	21–30	72 (34.29)	34 (25.56)	106 (30.90)		
	31–40	31 (14.76)	38 (28.57)	69 (20.12)		
	41–50	22 (10.48)	35 (26.32)	57 (16.62)		
	51–60	16 (7.62)	19 (14.29)	35 (10.20)		
	Above 61	2 (0.95)	3 (2.29)	5 (1.46)		
Education level	Informal	21 (10.00)	15 (11.28)	36 (10.50)	3	2.4594 (*p* = 0.483)
	Primary	75 (35.71)	57 (42.86)	132 (38.48)		
	Secondary	87 (41.43)	48 (36.09)	135 (39.36)		
	Tertiary	27 (12.86)	13 (9.77)	40 (11.6)		
Occupation	Hunters	111 (52.86)	14 (10.53)	125 (36.44)	5	88.8506 (*p* < 0.0001)
	Entrepreneurs	22 (10.48)	37 (27.82)	59 (17.20)		
	Farmers	41 (19.52)	63 (47.37)	104 (30.32)		
	Civil servant	7 (3.33)	11 (8.27)	18 (5.25)		
	Livestock keepers	1 (0.48)	3 (2.26)	4 (1.14)		
	Unemployed	28 (13.33)	5 (3.76)	33 (9.62)		
Site	Kapunga	72 (43.33)	21 (15.79)	112 (32.65)	2	122.7121 (*p* < 0.0001)
	Madibira	104 (49.52)	26 (19.55)	130 (37.90)		
	Utengule	15 (7.14)	86 (64.66)	101 (29.45)		

Additionally, another key informant shared the similar reasons for the declining trend of water bird population.

Although birds seem to be decreasing in the wetland, it is not easy to say to what extent such decrease has occurred due to lack of research data. But conversion of natural land to agriculture and other human uses might have generally reduced the suitable habitats and disturbed natural ecology of the wetland. (KI, male, Game officer)

### 3.4 Discussion

This study reveals variation in the relative abundance of water birds (Figure 1), which are likely attributable to the breeding success of particular species and habitat heterogeneity of the wetland. This finding is consistent with other related studies that reported that water birds' reproduction is much influenced by water levels, patch, and habitat size (Ma et al. 2009; Team R: Core 2018). Additionally, the uneven distribution of water birds among sites is probably due to the nature and characteristics of the microhabitats within each site. Large rice irrigation infrastructure in Kapunga, such as a permanent water reservoir, could be the reason for the observed aggregation of water birds' high abundance even when there is insufficient rainfall to flood the region. However, Utengule had a low abundance of waterfowl, most likely a result of frequent disturbances from livestock husbandry activities and less soil water holding capacity, hence less suitable for water birds. Furthermore, the abundance and distribution of water birds in Usangu wetland are invariably affected by distance from various land use/cover, rice extent growth, and habitat type. This study revealed that the abundance of water birds decreases with increasing distance from water bodies, albeit not significantly. This could be attributed to the period of sampling, March to May, where the plain is flooded and the existence of small pools of water in other land use types. This result differs from that of other studies in Chile and Ethiopia (Machibya and Mdemu 2005; Taherdoost 2017) where distance was an important factor determining the abundance and distribution of water birds. Likewise, the water birds' abundance increased along a distance gradient from settlement, grazing land, and farms (Table 2) but was not significant, probably because of the nucleated nature of settlement and grazing lands. Other studies found significance on the same variables contrary to these study findings (Team R: Core 2018).

Furthermore, rice farms provide a higher number of waterbirds in comparison to other habitats probably because they contain a higher amount of feed such as seeds, flies, beetles, snails, tadpoles, and small fishes. Similarly, studies show that the amount, composition, and spatio‐temporal distribution of food in rice farms are important for feeding and breeding for natives and act as stepping sites for long migratory water birds (IBM Corp N 2017). It is also argued that the presence of surface water in the rice fields and feed availability determine the richness and composition of water birds (Mengesha and Bekele 2008; Garay et al. 1991). On the other hand, open grasslands and maize farms hold fewer numbers of water birds as they are characterized by dry patches, less water holding capacity, and are frequently disturbed by livestock grazing. This is in agreement with other studies where habitat size, disturbance, and water level fluctuations are the most important variables affecting water birds distribution (González‐Gajardo et al. 2009). However, this finding contrasts with the results of the Brazilian study, which suggest that waterbirds abundance was higher in natural habitats in comparison to rice fields (Kicheleri and Ndibalema 2012).

Furthermore, the existence of small field paddies with high human disturbances hampers the reproductive output and feeding of birds in the Utengule and Madibira sites (Table 3). Similarly, rapid conversion of wetland to rice fields could have affected the small water birds including little stint, common snipe, and common sand piper which live in muddy pond edges, open habitats, and low vegetation cover (Elphick et al. 2010).

This study revealed that water use management and retention scale could be the key driving factors for variation in abundance and distribution of water birds between Kapunga rice schemes project and rice out‐growers (Table 2). Higher abundance of water birds observed in rice scheme project compared to farms of out‐growers may perhaps occurred because of well‐designed infrastructure of field paddies including small dams within the schemes, water canals and long period of water retention that provide suitable habitats for water birds. Amount and passability of water in irrigation farms may probably have positively attributed to the high density of water birds in schemes compared to out‐growers.

Cultivated areas showed higher abundance of waterbirds compared to uncultivated in the same habitats (Table 2) probably because of irrigation, which brings a sufficient amount of water supporting higher density of water birds. Nevertheless, despite the expansion of cultivated land being a key hotspot habitat for a number of water birds, it has been disadvantageous to breeding habitats of some species such as wandering whistling ducks and fulvous whistling ducks which breed out of the cultivated areas with long grasses. However, there is insufficient information on the scale of threats from breeding sites loss.

Moreover, the distribution of waterbirds varies significantly in field paddies depending on the extent and size of rice growth. Small waterbirds such as pied avocets, common ringed plovers, three banded plovers, little stints, and egrets are highly abundant in the vegetative stage of rice growth probably because it is open and does not inhibit them from walking and searching for food. Conversely, lesser moorhens and common moorhens' abundance was higher in grown rice (reproductive phase) compared to the vegetative phase possibly because they hide under tall dense rice crops and build their floating nests which prevent them from being harmed or threatened by predators. This study shows the importance of habitat heterogeneities as in rice farming as some birds such as gray herons, African open‐billed storks, gloss ibises, and marabou storks occupied in both extents of rice growth, but most were recorded in the levee of the vegetative phase. Despite moorhens' species being beneficiaries of long and dense vegetation cover (reproductive phase), it is destroyed during harvest season as the area becomes cleared for next season.

The social survey on population trends of water birds shows higher representation of males than females because most of the households' heads were males. The majority of the respondents being of active age could be disadvantageous in terms of conservation as they were mostly employed in unskilled jobs, which are not environmentally friendly. Nevertheless, 90% of the respondent's formal education (Table 4) may probably support conservation initiatives if the provided education is integrating conservation knowledge and skills (Fien et al. 2001). Local communities perceive a decline in bird abundance associated with age probably because it reflects experience differences and length of time accumulation of knowledge of the area and therefore a significant association (Tarakini et al. 2018). The significance of downward trend of water bird population in relation to type of occupation likely reflects experience with natural environment especially among hunters, farmers and livestock keeper compared to employees and entrepreneurs. In addition, significance in responses among sites could be attributed to differences in microhabitat variation and exposure of local communities to a higher assemblage of water birds into waterbirds.

## 4 Conclusion

This study concludes that the Usangu wetland has a high abundance of water birds with disproportionate distribution bias towards ice farms. In spite of rice farms suitably holding a high assemblage of water birds in the study area, they are artificial, changing after harvest and characterized by illegal hunting, which negatively affects the habitat of small water birds. The increasing conversion of land for rice farming leads to mortality, which may eventually result in the local disappearance of waterbirds and other species, hence threatening the integrity of the Usangu wetland.

## Author Contributions

**Amani Chaula:** conceptualization (equal), data curation (lead), formal analysis (equal), investigation (lead), methodology (equal), project administration (lead), visualization (equal), writing – original draft (equal), writing – review and editing (equal). **Safari Ignas:** conceptualization (equal), formal analysis (supporting), investigation (supporting), methodology (equal), writing – review and editing (equal). **Anibariki Ngonyoka:** conceptualization (lead), formal analysis (equal), methodology (equal), writing – original draft (equal), writing – review and editing (equal).

## Funding

The authors have nothing to report.

## Ethics Statement

This study was approved by the University of Dodoma's Institutional Research Review Committee (Ref: MA.84/261/02/‘A’/). Participants received full study information and provided informed consent prior to participation. They were guaranteed confidentiality, voluntary withdrawal rights, and data anonymization with secure storage per university policy.

## Conflicts of Interest

The authors declare no conflicts of interest.

## Supporting information


**Data S1:** ece373379‐sup‐0001‐supinfo.pdf.

**Data S2:** ece373379‐sup‐0002‐supinfo.xls.

**Data S3:** ece373379‐sup‐0003‐supinfo.xls.

**Data S4:** ece373379‐sup‐0004‐supinfo.dta.

**Data S5:** ece373379‐sup‐0005‐supinfo.txt.

**Data S6:** ece373379‐sup‐0006‐supinfo.do.

**Data S7:** ece373379‐sup‐0007‐supinfo.dta.

## Data Availability

The authors verify that the study's supporting data is accessible openly in the Mendeley data repository at https://doi.org/10.17632/4nw47pmd7x.2.
