# Supplementary material for: Abundance, Distribution and Population Trends of Waterbirds in the Usangu Wetland: A Biodiversity Hotspot Threatened by Human Activities
Source: Ecol Evol. 2026 Apr 8;16(4):e73379. doi: 10.1002/ece3.73379 (PMC13059676; doi:10.1002/ece3.73379)
Supplement: Supplementary file 1 — Data S1: ece373379‐sup‐0001‐supinfo.pdf. [file ECE3-16-e73379-s002.pdf]

Statistics/Data Analysis

1 . do "C:\Users\lenovo\AppData\Local\Temp\STD34c4_000000.tmp"

2 . use AMAN_DTA, clear

3 .
end of do-file

4 . do "C:\Users\lenovo\AppData\Local\Temp\STD34c4_000000.tmp"

5 . tabstat Noobserved, by(waterbirdspecies) s(N sum)

Summary for variables: Noobserved
by categories of: waterbirdspecies (water bird species)

waterbirdspecies	N	sum
African jacana	**4**	**10**
African openbill	**35**	**1073**
African sacred i	**18**	**589**
Black crake	**1**	**4**
Black stork	**27**	**98**
Black winged sti	**6**	**35**
Common greenshan	**4**	**1025**
Common moorhen	**19**	**1316**
Common ringed pl	**5**	**145**
Common sand pipe	**4**	**35**
Common snipe	**2**	**12**
Common-greenshan	**8**	**21**
Egyptian goose	**3**	**143**
Fulvious whistli	**30**	**3354**
Glossy ibis	**50**	**2836**
Great crested gr	**1**	**7**
Great egrets	**54**	**1754**
Grey heron	**59**	**205**
Greylag goose	**1**	**400**
Hammerkop	**2**	**4**
Intermediate egr	**6**	**62**
Little egrets	**38**	**746**
Little grebes	**1**	**20**
Little stint	**5**	**8**
Marabou stork	**5**	**13**
Pied Avocet	**4**	**17**
Reed cormorant	**8**	**137**
Saddle billed st	**4**	**14**
Three banded plo	**3**	**25**
White breasted w	**2**	**12**
White faced whis	**3**	**175**
White stork	**24**	**141**
Wondering whistl	**7**	**166**
Yellow billed st	**8**	**35**
great egrets	**1**	**3**
lesser moorhen	**26**	**13285**
white breasted w	**2**	**11**
Total	**480**	**27936**

6 . by location, sort : tabstat Noobserved, by(waterbirdspecies) s(N sum)

-> location = Madibira

Summary for variables: Noobserved
by categories of: waterbirdspecies (water bird species)

waterbirdspecies	N	sum
African jacana	3	9
African openbill	13	134
Black crake	1	4
Black stork	11	25
Black winged sti	4	21
Common moorhen	2	6
Common ringed pl	1	3
Common sand pipe	1	5
Common-greenshan	4	11
Egyptian goose	1	3
Fulvious whistli	10	158
Glossy ibis	22	295
Great egrets	32	1189
Grey heron	15	76
Little egrets	15	285
Little stint	2	3
Marabou stork	2	5
Three banded plo	1	3
White stork	6	14
Wondering whistl	3	100
Yellow billed st	3	8
great egrets	1	3
lesser moorhen	4	114
white breasted w	2	11
Total	159	2485

-> location = Utengule

Summary for variables: Noobserved
by categories of: waterbirdspecies (water bird species)

waterbirdspecies	N	sum
African openbill	14	534
African sacred i	7	479
Black stork	8	31
Black winged sti	1	2
Common sand pipe	2	10
Common snipe	1	5
Common-greenshan	4	10
Egyptian goose	1	20
Fulvious whistli	4	46
Glossy ibis	9	414
Great egrets	3	48
Grey heron	24	65
Intermediate egr	1	4
Little egrets	10	307
Little grebes	1	20
Little stint	3	5
Marabou stork	2	6
Pied Avocet	1	2
Reed cormorant	2	16
Saddle billed st	2	4
White breasted w	2	12
White stork	15	46
Wondering whistl	2	36
Yellow billed st	3	17
lesser moorhen	5	21
Total	127	2160

-> location = kapunga

Summary for variables: Noobserved
by categories of: waterbirdspecies (water bird species)

waterbirdspecies	N	sum
African jacana	1	1
African openbill	8	405
African sacred i	11	110
Black stork	8	42
Black winged sti	1	12
Common greenshan	4	1025
Common moorhen	17	1310
Common ringed pl	4	142
Common sand pipe	1	20
Common snipe	1	7
Egyptian goose	1	120
Fulvious whistli	16	3150
Glossy ibis	19	2127
Great crested gr	1	7
Great egrets	19	517
Grey heron	20	64
Greylag goose	1	400
Hammerkop	2	4
Intermediate egr	5	58
Little egrets	13	154
Marabou stork	1	2
Pied Avocet	3	15
Reed cormorant	6	121
Saddle billed st	2	10
Three banded plo	2	22
White faced whis	3	175
White stork	3	81
Wondering whistl	2	30
Yellow billed st	2	10
lesser moorhen	17	13150
Total	194	23291

7 . by Habitattype, sort : tabstat Noobserved, by(waterbirdspecies) s(N sum)

-> Habitattype = Rice farm

Summary for variables: Noobserved
by categories of: waterbirdspecies (water bird species)

waterbirdspecies	N	sum
African jacana	2	4
African openbill	28	956
African sacred i	15	581
Black crake	1	4
Black stork	25	93
Black winged sti	5	23
Common greenshan	2	1000
Common moorhen	18	1216
Common ringed pl	3	38
Common sand pipe	3	32
Common snipe	2	12
Common-greenshan	8	21
Fulvious whistli	24	2320
Glossy ibis	49	2776
Great egrets	51	1716
Grey heron	51	185
Hammerkop	2	4
Intermediate egr	4	53
Little egrets	32	685
Little stint	4	7
Marabou stork	4	11
Pied Avocet	1	2
Reed cormorant	4	51

Saddle billed st	4	14
White faced whis	2	145
White stork	22	136
Wondering whistl	4	105
Yellow billed st	8	35
lesser moorhen	26	13285
white breasted w	2	11
Total	406	25521

-> Habitattype = grassland

Summary for variables: Noobserved
by categories of: waterbirdspecies (water bird species)

waterbirdspecies	N	sum
African openbill	4	44
African sacred i	3	8
Black stork	2	5
Great egrets	1	3
Grey heron	7	14
Intermediate egr	1	4
Little egrets	5	31
Little stint	1	1
White breasted w	2	12
White stork	1	1
Wondering whistl	1	6
Total	28	129

-> Habitattype = maizefarm

Summary for variables: Noobserved
by categories of: waterbirdspecies (water bird species)

waterbirdspecies	N	sum
African openbill	2	40
Common sand pipe	1	3
Great egrets	1	15
Little egrets	1	30
White stork	1	4
Total	6	92

-> Habitattype = water pond

Summary for variables: Noobserved
by categories of: waterbirdspecies (water bird species)

waterbirdspecies	N	sum
African jacana	2	6
African openbill	1	33
Black winged sti	1	12
Common greenshan	2	25
Common moorhen	1	100
Common ringed pl	2	107
Egyptian goose	3	143
Fulvious whistli	6	1034
Glossy ibis	1	60
Great crested gr	1	7
Great egrets	1	20
Grey heron	1	6
Greylag goose	1	400

Intermediate egr	1	5
Little grebes	1	20
Marabou stork	1	2
Pied Avocet	3	15
Reed cormorant	4	86
Three banded plo	3	25
White faced whis	1	30
Wondering whistl	2	55
great egrets	1	3
Total	40	2194

8 . by Habitattype, sort : tabstat Noobserved, by(waterbirdspecies) s(N sum)

-> Habitattype = Rice farm

Summary for variables: Noobserved
by categories of: waterbirdspecies (water bird species)

waterbirdspecies	N	sum
African jacana	2	4
African openbill	28	956
African sacred i	15	581
Black crake	1	4
Black stork	25	93
Black winged sti	5	23
Common greenshan	2	1000
Common moorhen	18	1216
Common ringed pl	3	38
Common sand pipe	3	32
Common snipe	2	12
Common-greenshan	8	21
Fulvious whistli	24	2320
Glossy ibis	49	2776
Great egrets	51	1716
Grey heron	51	185
Hammerkop	2	4
Intermediate egr	4	53
Little egrets	32	685
Little stint	4	7
Marabou stork	4	11
Pied Avocet	1	2
Reed cormorant	4	51
Saddle billed st	4	14
White faced whis	2	145
White stork	22	136
Wondering whistl	4	105
Yellow billed st	8	35
lesser moorhen	26	13285
white breasted w	2	11
Total	406	25521

-> Habitattype = grassland

Summary for variables: Noobserved
by categories of: waterbirdspecies (water bird species)

waterbirdspecies	N	sum
African openbill	4	44
African sacred i	3	8
Black stork	2	5
Great egrets	1	3
Grey heron	7	14
Intermediate egr	1	4
Little egrets	5	31
Little stint	1	1
White breasted w	2	12
White stork	1	1
Wondering whistl	1	6
Total	28	129

-> Habitattype = maizefarm

Summary for variables: Noobserved
by categories of: waterbirdspecies (water bird species)

waterbirdspecies	N	sum
African openbill	2	40
Common sand pipe	1	3
Great egrets	1	15
Little egrets	1	30
White stork	1	4
Total	6	92

-> Habitattype = water pond

Summary for variables: Noobserved
by categories of: waterbirdspecies (water bird species)

waterbirdspecies	N	sum
African jacana	2	6
African openbill	1	33
Black winged sti	1	12
Common greenshan	2	25
Common moorhen	1	100
Common ringed pl	2	107
Egyptian goose	3	143
Fulvious whistli	6	1034
Glossy ibis	1	60
Great crested gr	1	7
Great egrets	1	20
Grey heron	1	6
Greylag goose	1	400
Intermediate egr	1	5
Little grebes	1	20
Marabou stork	1	2
Pied Avocet	3	15
Reed cormorant	4	86
Three banded plo	3	25
White faced whis	1	30
Wondering whistl	2	55
great egrets	1	3
Total	40	2194

```
9 .
10. *log using Amani_table2.smcl, replace
11. by location, sort : tabstat Noobserved, by(Schemeoutgrowers) s(N sum)
```

-> location = Madibira

Summary for variables: Noobserved
by categories of: Schemeoutgrowers (Scheme/outgrowers)

Schemeoutgrowers	N	sum
O.G	**159**	**2485**
Total	**159**	**2485**

-> location = Utengule

Summary for variables: Noobserved
by categories of: Schemeoutgrowers (Scheme/outgrowers)

Schemeoutgrowers	N	sum
O.G	**127**	**2160**
Total	**127**	**2160**

-> location = kapunga

Summary for variables: Noobserved
by categories of: Schemeoutgrowers (Scheme/outgrowers)

Schemeoutgrowers	N	sum
O.G	**75**	**874**
Scheme	**119**	**22417**
Total	**194**	**23291**

```
12. by location, sort : tabstat Noobserved, by(Ricegrowthextent) s(N sum)
```

-> location = Madibira

Summary for variables: Noobserved
by categories of: Ricegrowthextent (Rice growth extent)

Ricegrowthextent	N	sum
Vegetative phase	**159**	**2485**
Total	**159**	**2485**

-> location = Utengule

Summary for variables: Noobserved
by categories of: Ricegrowthextent (Rice growth extent)

Ricegrowthextent	N	sum
Vegetative phase	97	1952
no field paady	30	208
Total	127	2160

-> location = kapunga

Summary for variables: Noobserved
by categories of: Ricegrowthextent (Rice growth extent)

Ricegrowthextent	N	sum
Reproductive pha	116	21410
Vegetative phase	71	1852
no field paady	7	29
Total	194	23291

```
13. *log close
14.
15. reg Noobserved DWaterbodiesm DSettlementm Dgrazingblandm DFarmm i.Ricegrowthextent_cat ib3.lo
> cation_cat i.Habitattype_cat, rob
```

Linear regression

Number of obs	=	479
F(11, 467)	=	6.26
Prob > F	=	0.0000
R-squared	=	0.1910
Root MSE	=	154.77

Noobserved	Coef.	Robust Std. Err.	t	P>|t|	[95% Conf.	Interval]
DWaterbodiesm	-.0372291	.0190953	-1.95	0.052	-.0747524	.0002943
DSettlementm	-.0046919	.0100822	-0.47	0.642	-.0245039	.0151202
Dgrazingblandm	.0099104	.0053744	1.84	0.066	-.0006506	.0204714
DFarmm	.0448459	.0350021	1.28	0.201	-.0239352	.1136269
Ricegrowthextent_cat						
Vegetative phase	-162.7596	30.97951	-5.25	0.000	-223.6361	-101.8831
no field paady	-105.6484	25.38603	-4.16	0.000	-155.5334	-55.76338
location_cat						
Madibira	-16.43562	11.85681	-1.39	0.166	-39.73492	6.86368
Utengule	-2.735815	10.48827	-0.26	0.794	-23.34587	17.87424
Habitattype_cat						
grassland	-71.31577	28.27526	-2.52	0.012	-126.8783	-15.75328
maizefarm	-61.90463	29.1416	-2.12	0.034	-119.1695	-4.639733
water pond	-70.59503	27.80974	-2.54	0.011	-125.2427	-15.94732
_cons	179.4781	31.64179	5.67	0.000	117.3002	241.656

```
16. outreg2 using Reg222OG, word ctitle("General")
Reg222OG.rtf
dir : seeout
```

```
17. reg Noobserved i.Cultivationstaus_cat ib3.location_cat, rob

  Linear regression                               Number of obs     =        480
                                                  F(3, 476)         =      12.40
                                                  Prob > F          =     0.0000
                                                  R-squared         =     0.0934
                                                  Root MSE          =      162.3
```

Noobserved	Coef.	Robust Std. Err.	t	P>|t|	[95% Conf.	Interval]
Cultivationstaus_cat						
X	-42.10233	10.08688	-4.17	0.000	-61.92264	-22.28202
location_cat						
Madibira	-105.9469	18.40739	-5.76	0.000	-142.1167	-69.77715
Utengule	-96.61164	17.857	-5.41	0.000	-131.6999	-61.52334
_cons	121.5759	18.06186	6.73	0.000	86.08502	157.0667

```
18. outreg2 using Reg222OG, word append ctitle("Cultivation")
  Reg222OG.rtf
  dir : seeout

19. reg Noobserved i.Schemeoutgrowers_cat if location=="kapunga", rob

  Linear regression                               Number of obs     =        194
                                                  F(1, 192)         =      41.29
                                                  Prob > F          =     0.0000
                                                  R-squared         =     0.1196
                                                  Root MSE          =     234.75
```

Noobserved	Coef.	Robust Std. Err.	t	P>|t|	[95% Conf.	Interval]
Schemeoutgrowers_cat						
Scheme	176.7248	27.50106	6.43	0.000	122.4818	230.9678
_cons	11.65333	1.485633	7.84	0.000	8.723075	14.58359

```
20. outreg2 using Reg222OG, word append ctitle("Schme_kapunga")
  Reg222OG.rtf
  dir : seeout

21.
  end of do-file

22. translate @Results Amani_outputs.pdf, replace
```
